# Effect of m^6^A RNA Methylation Regulators on Malignant Progression and Prognosis in Renal Clear Cell Carcinoma

**DOI:** 10.3389/fonc.2020.00003

**Published:** 2020-01-24

**Authors:** Jiawu Wang, Chengyao Zhang, Weiyang He, Xin Gou

**Affiliations:** ^1^Department of Urology, The First Affiliated Hospital of Chongqing Medical University, Chongqing, China; ^2^Department of Oral Maxillofacial-Head and Neck Oncology, Shanghai Ninth People's Hospital, College of Stomatology, Shanghai Jiao Tong University School of Medicine, Shanghai, China; ^3^Department of Head and Neck Cancer Center, Chongqing University Cancer Hospital & Chongqing Cancer Institute & Chongqing Cancer Hospital, Chongqing, China

**Keywords:** m^6^A methylation, methyltransferase, demethylases, epigenetics, prognostic signature

## Abstract

**Objectives:** This study aims to explore the roles of 13 m^6^A RNA methylation regulators in clear cell renal cell carcinoma (ccRCC), and identify a risk signature and prognostic values of m^6^A RNA methylation regulators in ccRCC.

**Materials and Methods:** RNA sequence data of ccRCC was obtained from The Cancer Genome Atlas (TCGA) database. Differentially expressed of 13 m^6^A RNA methylation regulators in ccRCC stratified by different clinicopathological characteristics were unveiled using “limma” package in R version 3.6.0. Cox regression and LASSO analyses were conducted to identify the powerful independent prognostic factors in ccRCC associated with overall survival (OS). Protein-protein interaction (PPI) network and correlation analyses of the 13 m^6^A RNA methylation regulators were performed using “STRING” and R package, respectively. Principal component analysis (PCA) was also done using R. In addition, gene ontology (GO), GSEA and Kyoto Encyclopedia of Genes and Genomes pathways were used to functionally annotate the differentially expressed genes in different subgroups.

**Results:** Most of the 13 m^6^A RNA methylation regulators are differentially expressed in ccRCC tissue samples stratified by different clinicopathological characteristics in 537 patients. Next, a risk signature for predicting prognosis of ccRCC patients, was established based on two powerful independent prognostic m^6^A RNA methylation regulators (METTL14 and METTL3). Then, two subgroups (cluster1 and 2) were identified by consensus clustering to the two powerful independent factors and the cluster1 had a poorer prognosis than cluster2. Furthermore, the genes in cluster1 were significantly enriched in cancer-related pathways, biological process, and hallmarks, including “cell adhesion molecules (CAMs),” “leukocyte migration,” “Wnt/β-catenin signaling,” and so on.

**Conclusion:** M^6^A RNA methylation regulators play important roles in the initiation and progression of ccRCC and provide a novel sight to understand m^6^A RNA modification in ccRCC.

## Introduction

Renal cell carcinoma (RCC) is one of the most common adult genitourinary cancer, with ~73,820 newly diagnosed cases and nearly 14,770 of RCC-related mortality in the United States (1). Clear cell renal cell carcinoma (ccRCC) is the most common histological subtype of RCC, account for 70% (2). Although the diagnostic techniques of renal cell carcinoma are improved in the past decades, ~30% ccRCC patients have already developed metastases or local progression at the diagnosis (3). As ccRCC resistance to radiotherapy and chemotherapy, surgery is still an effective method for the treatment of ccRCC at present (4, 5). However, around one third of patients with localized RCC experience local recurrence and metastasis after surgical treatment (6). Complicated biological processes and unintelligible molecular mechanism lead to unfavorable prognosis of ccRCC patients. Hence, there is an urgent need to explore the molecular mechanism of ccRCC and identify novel targets for treatment and intervention for ccRCC patients.

Currently, the functions of RNAs in various cellular processer attract more and more attentions and accumulating studies are emerged in this field during the past decade. Up till now, many chemical modifications have been identified in distinct types of RNAs, including tRNA, miRNA, mRNA, long non-coding RNA, and others (7–12). These RNA modifications are reported to be several forms, such as N7-methyladenosine, 5-methylcytosine, N6-methyladenosine (m^6^A), and 2′-O-methylaion (7, 11, 13). M^6^A modification has been proved to be the most widespread, abundant and conserved form of mRNA methylation in eukaryotes (11, 14, 15). Generally speaking, m^6^A is enriched in 3′untranslated terminal region (UTR), near long internal exons and stop codons (16), thereby leading to alterations of RNA transcription, translation, metabolism, and processing (7, 17–20). As we known that RNA modification is mediated by a methyltransferase complex associated with three homologous factors named “readers” (YTHDC1, YTHDC2, YTHDF1, YTHDF2, and HNRNPC), “writers” (METTL3, METTL14, WTAP, KIAA1429, RBM15, and ZC3H13) and “erasers” (FTO and ALKBH5) (8, 21–26). M^6^A RNA methylation exhibits a reversible and dynamic biological process through the regulation of writers, readers and erasers. The identification of the m^6^A RNA methylation regulators bring us a new insight for the role of m^6^A modification in the regulation of gene expression (12, 27, 28).

Accumulating evidences have suggested the fact that the dysregulated expression of m^6^A RNA methylation regulators are involved in the initiation and development of human cancers. It was reported that METTL3, induced by hepatitis B X-interacting protein, could facilitate the proliferation of breast cancer through inhibiting the expression of let-7g (29). High expression of YTHDF1 was found to be associated with advanced stages and unfavorable prognosis in hepatocellular carcinoma (30). The key role of m^6^A RNA methylation regulators are also noted in different cancers, such as lung cancer (31), bladder cancer (32), and nasopharyngeal carcinoma (33). Recently, a research focused on the mutations and copy number variants of 10 m^6^A regulatory genes in ccRCC and found copy number variants of these regulatory genes closely correlated with pathologic stage and prognosis of patients with ccRCC (34). However, there is still a lack of comprehensive analyses of m^6^A RNA methylation modification in ccRCC with various clinicopathological features.

In the present study, we assessed the correlation of the expression levels of 13 m^6^A RNA regulators and clinicopathological characteristic in ccRCC based on the data obtained from The Cancer Genome Atlas (TCGA) (*n* = 537). We discovered that m^6^A RNA methylation regulators served as important roles in the initiation and progression of ccRCC, and according to two powerful independent prognostic m^6^A methylation regulators, a risk signature was established to classify the prognosis of ccRCC.

## Materials and Methods

### Study Cohort

RNA sequence data from 537 patients with ccRCC were retrieved from The Cancer Genome Atlas (TCGA) database (https://cancergenome.nih.gov/) in 2019 and the values of these RNA sequence data were normalized by expectation-maximization. Patient clinical information was obtained using the Data Transfer Tool (provided by GDC Apps) (TCGA sample IDs and RNA-Seq information were presented in Supplementary Table 1). Clinicopathological information for the 537 ccRCC patients was summarized in Table 1. This study met the publication guidelines stated by TCGA (https://cancergenome.nih.Gov/publications/publicationguidelines). All data used in the study was obtained from TCGA, and hence ethics approval and informed consent were not required.

**Table 1:** Clinicopathological features of patients included in this study.

	**Total patients (537)**		**High-risk group (262)**		**Low-risk group (263)**		***p*-value**
	**Number**	**Percentage (%)**	**Number**	**Percentage (%)**	**Number**	**Percentage(%)**	
Age							9.44E-02
≤60	266	49.5	129	49.2	135	51.3	
>60	271	50.5	133	50.8	128	48.7	
Gender							1.89E-01
Female	191	35.6	81	30.9	101	38.4	
Male	346	64.4	181	69.1	162	61.6	
Grade							4.72E-04
G1	14	2.6	7	2.7	6	2.3	
G2	230	42.8	106	40.5	120	45.6	
G3	207	38.5	104	39.7	100	38.0	
G4	78	14.5	41	15.6	33	12.5	
Gx	5	0.9	2	0.8	3	1.1	
Unknown	3	0.6	2	0.8	1	0.4	
Stage							2.15E-04
I	269	50.1	135	51.5	126	47.9	
II	57	10.6	24	9.2	32	12.2	
III	125	23.3	61	23.3	62	23.6	
IV	83	15.5	40	15.3	42	16.0	
Unknown	3	0.6	2	0.8	1	0.4	
Stage T							4.66E-04
T1	275	51.2	139	53.1	128	48.7	
T2	69	12.8	32	12.2	36	13.7	
T3	182	33.9	85	32.4	94	35.7	
T4	11	2.0	6	2.3	5	1.9	
Stage M							2.06E-03
M0	426	79.3	210	80.2	207	78.7	
M1	79	14.7	39	14.9	39	14.8	
Mx	30	5.6	11	4.2	17	6.5	
Unknown	2	0.4	2	0.8		0.0	
Stage N							5.40E-02
N0	240	44.7	114	43.5	123	46.8	
N1	17	3.2	9	3.4	7	2.7	
Nx	280	52.1	139	53.1	133	50.6	

### Selection of m^6^A RNA Methylation Regulators

According to study published by Yang Y et al. (8), 13 m^6^A RNA methylation regulators, including ALKBH5, FTO, YTHDC1, YTHDC2, YTHDF1, YTHDF2, HNRNPC, METTL3, METTL14, WTAP, KIAA1429, RBM15, and ZC3H13, were used for our analysis. Then, the correlation between the expression of these m^6^A RNA methylation regulators and different clinicopathological characteristics were evaluated based on the data from TCGA.

### Bioinformatic Analysis

**Figure 1:**
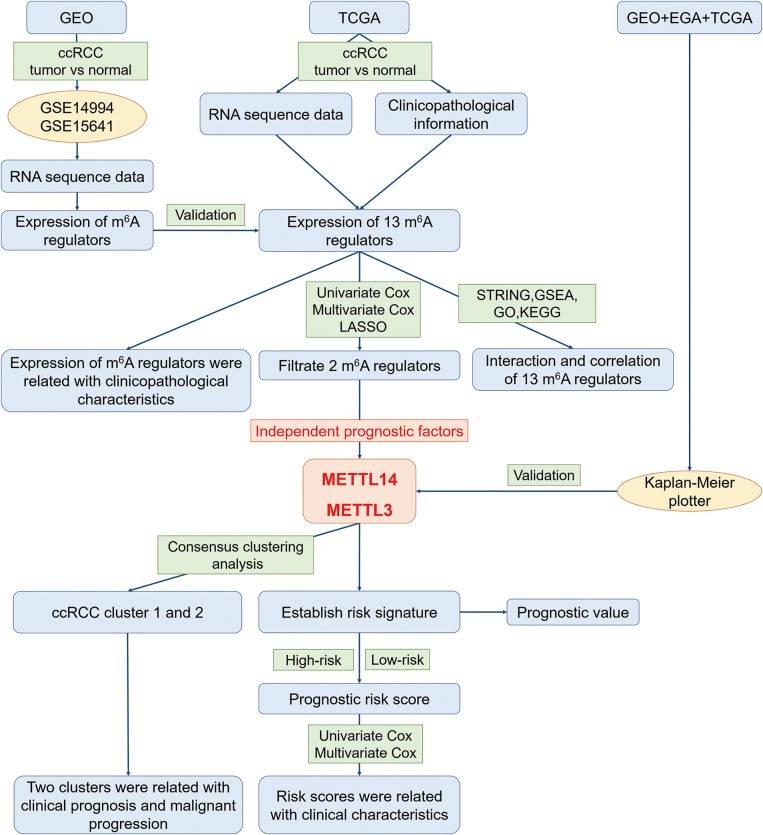
The flow chart of the study design and analysis.

In order to explore the roles of the m^6^A RNA methylation regulators in ccRCC, our study was designed and analyzed according to the flow chart (Figure 1). The relationship of expressions of these 13 regulators and distinct clinicopathological characteristics in ccRCC was analyzed using the “limma” package (http://www.bioconductor.org/packages/release/bioc/html/limma.html) with a cut-off criteria of *p* < 0.05. Then, the differentially expressed m^6^A methylation regulators between tumor tissues and normal tissues were verified by two gene expression profiles (GSE14994 and GSE15641), which were downloaded from Gene Expression Omnibus (GEO) database (https://www.ncbi.nlm.nih.gov/geo/). The GSE14994 datasets included 59 ccRCC samples and 11 normal samples, while GSE5641 datasets contained 32 ccRCC samples and 23 normal samples. The differentially expressed m^6^A methylation regulators between tumor tissues and normal tissues was also analyzed using the “limma” package (http://www.bioconductor.org/packages/release/bioc/html/limma.html) with a cut-off criteria of *p* < 0.05. Next, a PPI network of the 13 m^6^A RNA methylation regulators was constructed by using the Search Tool for the Retrieval of Interacting Genes (STRING, http://string.embl.de/). The combined score higher than 0.70 was regarded statistical significance. The correlation analysis was also performed by R package. Then, the prognostic m^6^A RNA methylation regulators were identified using univariate Cox regression analysis. Based on the results of univariate analysis, seven regulators highly correlated with overall survival (OS) (*p* < 0.05) were selected for the LASSO Cox regression analysis (35). Then, 2 m^6^A regulators were identified as the powerful independent prognostic factors by LASSO analysis. In addition, the prognostic values of these two regulators were verified by the Kaplan Meier plotter (www.kmplot.com), an online tool based on Gene Expression Omnibus database (GEO), European Genome-phenome Archive (EGA), and TCGA (36). The hazard ratio (HR) with 95% confidence intervals and log-rank *P*-value were calculated. Log-rank *p* > 0.05 was considered statistical significance. HR > 1, gene expression was negatively associated with OS., while HR < 1, gene expression was positively associated with OS. At last, two regulators' coefficients were identified according to the best penalty parameter λ. The risk score (RS) was estimated using the following formula:

$$\begin{array}{l} RS=\overset{n}{\underset{i=1}{\sum }}\text{Coef}\left(i\right)X\left(i\right) \end{array}$$

where *n* represents the number of modules RNAs; Coef (i) d is the coefficient; X(i) denotes the z-score-transformed relative expression level for each gene identified by LASSO analysis. When the RS for a given sample was less than the mean RS of all samples, the latter was considered the low risk sample, otherwise, it was considered a high-risk sample. Survival curves in the high-risk and low-risk groups were estimated using the Kaplan-Meier method. Additionally, the receiver operating characteristic (ROC) curves and area under the ROC curves (AUC values) were used to access sensitivity and specificity.

Furthermore, two different subgroups (cluster1 and 2) were identified using “Consensus ClusterPlus” package (https://www.bioconductor.org/packages/release/bioc/html/ConsensusClusterPlus.html) with resample rate of 80%, 50 iterations and Pearson correlation. Principal component analysis (PCA) was performed by the R package for R version 3.6.0 to assess the gene expression patterns in the two ccRCC subgroups. Gene ontology (GO) and Kyoto Encyclopedia of Genes and Genomes (KEEG) pathway analyses were done with the R packages in R version 3.6.0 to functionally annotate the differentially expressed genes in different subgroups. Gene Set Enrichment Analysis (GSEA) was also conducted to study the functions associated with different subgroups of ccRCC. Hallmark gene set “h.all.v6.0.symbol.gmt” was applied in GSEA analysis.

### Statistical Analysis

The *t*-test was performed to investigate the distribution of risk score in patients grouped by grade or classification. Univariate and multivariate Cox regression analyses were applied to identified the prognostic factors and different clinicopathological characteristics. Survival curves was plotted by using the “survival” package in R. Long-rank test was used to assess statistical significance. All statistical results with *p* < 0.05 were regard to be statistically significant.

## Results

### Expression of m^6^A RNA Methylation Regulators Is Associated With Clinicopathological Characteristics in ccRCC

**Figure 2:**
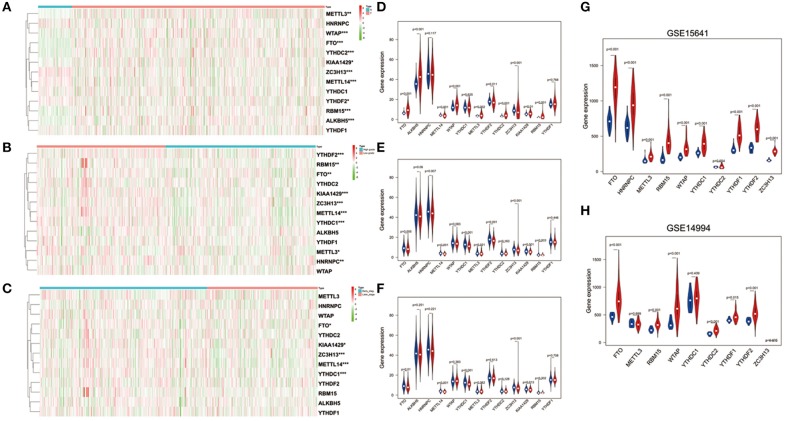
Expression of m^6^A RNA methylation regulators in ccRCC with different clinicopathological characteristics. **(A–C)** The heatmaps of 13 m^6^A RNA methylation regulators in different tissue samples (**A** for tumor status, **B** for WHO grade, and **C** for pathological stage). **(D–F)** The expression of 13 m^6^A RNA methylation regulators in different tissue samples (**D** for tumor status, **E** for WHO grade, and **F** for pathological stage). **(G,H)** The expression of m^6^A RNA methylation regulators in tumor tissues and normal tissues (**G** for GSE15641 and **H** for GSE14994). *p < 0.05, **p < 0.01, and ***p < 0.001; the red fusiformis represents tumor tissue and the blue fusiformis represents normal tissue.

To better understand the important roles of m^6^A RNA methylation regulators in oncogenesis and progression, we explored expression levels of m^6^A RNA methylation regulators in different tissue samples, including tumor status (normal and tumor), WHO grade [low grade (including Grade I and II) and high grade (including Grade III and IV)], as well as pathological stage (early stage including Stage I and II, later stage including Stage III and IV) tissue samples. The results are shown as heatmaps in Figures 2A–C, suggesting that the expressions of most m^6^A methylation regulators are significantly related to tumor status. Ten m^6^A RNA methylation regulators were significantly abnormally expressed in ccRCC tissues samples. Compared to normal tissue samples, ZC3H13, METTL14, and YTHDF2 were down regulated, while FTO, ALKBH5, WTAP, METTL3, YTHDC2, KIAA1429, and RBM15 were up regulated in ccRCC tissue samples (Figure 2D). These differentially expressed regulators were verified by GSE15641 and GSE15641 datasets. The results of validation suggested that FTO, WTAP, RBM15, and ZC3H13 were significantly abnormally expressed in ccRCC tissues (Figures 2G,H), which was consistent with our previous result. However, ZC3H13 was down regulated in ccRCC samples in GSE14994 datasets while the opposite result was observed in ccRCC samples in both GSE15641 datasets and TCGA. This may be due to the different study populations and different scales.

Next, we investigated the relationship between expression of m^6^A RNA methylation regulators, WHO grade (Figure 2E) and pathological stage (Figure 2F) in ccRCC, respectively. The results demonstrated that low expression levels of FTO, METTLE14, YTHDC1, ZC3H13, and KIAA1429 were significantly associated with both high grade and later pathological stage. Besides that, HNRNPC, METTL3, YTHDF2, and RBM15 were also observed down regulation in high grade tissue samples.

### Interaction and Correlation of 13 m^6^A RNA Methylation Regulators in ccRCC

**Figure 3:**
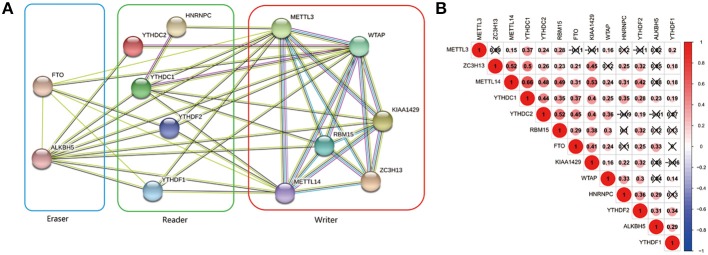
Interaction among m^6^A RNA methylation regulators. **(A)** The PPI network of the 13 m^6^A methylation regulators constructed using STRING. **(B)** Spearman correlation analysis of the 13 m^6^A methylation regulators.

The interrelationships between the 13 m^6^A RNA methylation genes were retrieved from STRING database to construct PPI network (Figure 3A) and their correlations were also analyzed using “corrplot” package in R software (Figure 3B). As a result, we found that there were close and complicated interrelationships between the six writers (Figure 3A). The expressions of the six writers were significantly associated with each other except METTL3 and ZC3H13, METTL3 and KIAA1429, ZC3H13 and WTAP in ccRCC (Figure 3B). Few interactions were observed between the five readers in PPI network (Figure 3A). However, the significant correlations between HNRNPC, YTHDC2, YTHDC1, YTHDF1, and YTHDF2 were observed in ccRCC (Figure 3B). There were evidences supporting the interaction between FTO and ALKBH5 in PPI network (Figure 3A), and the expressions of FTO and ALKBH5 were positively associated with each other in ccRCC (Figure 3B). In addition, the expressions of FTO, KIAA1429, ZC3H13, METTL14, and YTHDC1 were highly related to each other (Figure 3B), which agreed with that the expressions of these genes mentioned above being negatively associated with the increasing malignancy of ccRCC.

### Prognostic Value of m^6^A RNA Methylation Regulators and a Risk Signature Established Based on Two Identified m^6^A RNA Methylation Regulators

**Figure 4:**
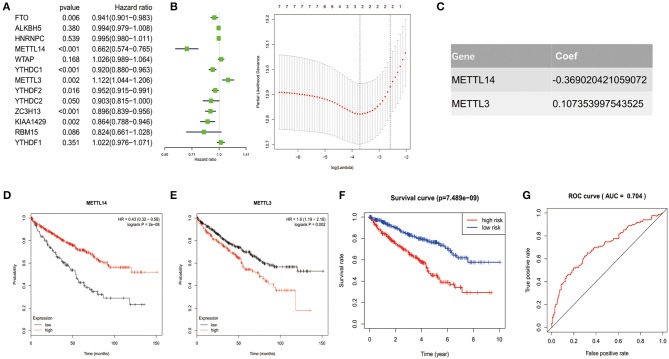
Risk signature with 2 m^6^A RNA methylation regulators. **(A–C)** The process of constructing the signature based on 2 m^6^A RNA methylation regulators. The Hazard ratio (HR), 95% confidence interval (CI) estimated by univariate Cox regression **(A)** and the coefficients estimated by multivariate Cox regression via LASSO are presented **(B,C)**. **(D,E)** The overall survival curves of the two regulators estimated by the Kaplan Meier plotter. Log-rank *p* < 0.05 was considered statistical significance. HR > 1, gene expression was negatively associated with OS., while HR < 1, gene expression was positively associated with OS. **(F)** Kaplan-Meier overall survival (OS) curves for patients in high- and low-risk group divided according to the risk score. **(G)** ROC analysis and AUC value of the ROC curve suggesting the sensitivity and specificity for risk signature.

In order to investigate the prognostic value of these 13 m^6^A RNA methylation regulators in ccRCC, univariate Cox regression analysis was performed based on the expression levels of the regulators from TCGA (Figure 4A). As a result, we found that seven out of the 13 regulators were significantly associated with overall survival (OS) (*p* < 0.05). Among the seven regulators, only METTL3 was considered as risky gene with HR> 1, while FTO, METTL14, YTHDC1, YTHDC2, ZC3H13, and KIAA1429 were considered as protective genes with HR <1.

To identified the most powerful prognostic m^6^A RNA methylation regulators, the last absolute shrinkage and selection operator (LASSO) Cox regression analysis to the seven prognosis-related genes was conducted (Figure 4B) and the coefficient of each independent prognostic gene was shown in Figure 4C. The LASSO results showed that two regulators (METTL14 and METTL3) were the powerful prognostic factors. Then, to verify the two regulators, their prognostic values were verified by the Kaplan Meier plotter. The results showed that ccRCC patients with high METTL14 expression had favorable prognosis (Figure 4D), while ccRCC patients with high METTL3 expression had bad prognosis (Figure 4E), which was in accordance with the LASSSO results and this partly strength the reliability of our findings.

Based on the powerful prognostic factors (METTL14 and METTL3), a risk signature was constructed. Then, the risk score was estimated based on the coefficients obtained from the LASSO analysis. In order to test the prognostic role of the two-gene risk signature, ccRCC patients from TCGA (*n* = 525) were assigned into high-risk and low-risk groups according to the median risk score (Table 1). The result of survival analysis showed that the high-risk group had significantly shorter survival time compared to low-risk group (Figure 4F). The 5-years OS was 44.3% in high-risk group and 75.2% in low-risk group. Time-dependent ROC curve was used to assess the sensitivity and specificity of the prediction and the result showed that AUC values was 0.704 (Figure 4G), suggesting well-prediction performances.

### Prognostic Risk Score Indicated Strong Associations With Clinical Characteristics in ccRCC

**Figure 5:**
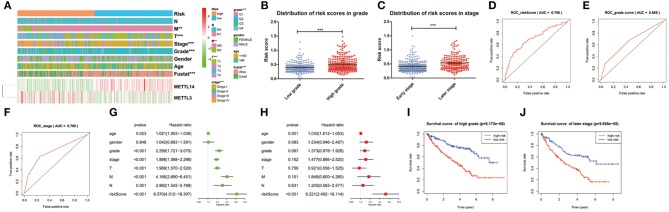
Relation between the risk score and clinicopathological characteristics, as well as prognostic value of the risk signature in patients stratified by WHO grade and pathological stage. **(A)** The heatmap shows the expression of the 2 m^6^A RNA methylation regulators in high-risk and low-risk ccRCC. The distribution of clinicopathological characteristics was compared between the high-risk and low-risk groups. *p < 0.05, **p < 0.01 and ***p < 0.001. **(B,C)** The distribution of risk score in patients stratified by WHO grade and pathological stage. *p < 0.05, **p < 0.01, and ***p < 0.001. **(D–F)** ROC curves showed the predictive efficiency of the risk score **(D)**, WHO grade **(E)**, and pathological stage **(F)**. **(G)** Univariate Cox regression analysis of the associated between clinicopathological factors (including risk score) and overall survival of patients. **(H)** Multivariate Cox regression analysis of the associated between clinicopathological factors (including risk score) and overall survival of patients. **(I)** Kaplan-Meier overall survival curve for patients with low grade and high grade. **(J)** Kaplan-Meier overall survival curve for patients with early grade and later pathological stage.

The expression levels of the two identified m^6^A RNA methylation regulators in high-risk and low-risk group were presented in the heatmap (Figure 5A). The results showed that there were significant differences between the high-risk and low-risk groups in term of grade (*p* < 0.001), pathological stage (*p* < 0.001), stage T (*p* < 0.001), and stage M (*p* < 0.01). The relationship between the risk score and each clinicopathological characteristics was also explored in the present study. The results showed that the risk score has significant differences between patients divided by WHO grade and pathological stage (Figures 5B,C). Furthermore, the ROC curve showed that the risk score (AUC = 0.708) (Figure 5D) was better than grade (AUC = 0.655) in predicting 5-years survival rate (Figure 5E) and was similar to pathological stage (AUC = 0.709) in prediction for 5-years survival rate (Figure 5F).

To test whether the risk signature was an independent prognostic factor, univariate and multivariate Cox regression analyses were performed. As a result, the age at diagnosis, grade, pathological stage, stage TNM and risk score were associated with OS in univariate analysis (Figure 5G) and only risk score and age at diagnosis were still significantly related to OS (*p* < 0.05) in multivariate Cox regression analysis (Figure 5H). The prognostic value of the risk signature for different grades and pathological stage was also analyzed using Kaplan-Meier curve. The results showed that patients with low risk scores had a significantly longer survival time than those with high scores in high grade (Figure 5I) and later stage (Figure 5J). These results demonstrated that the risk score retrieved from m^6^A RNA methylation regulators could be served as an independent prognostic factor in ccRCC.

### Consensus Clustering of Two Independent Prognostic m^6^A RNA Methylation Regulators Identified Two Clusters of ccRCC With Different Clinical Outcomes

**Figure 6:**
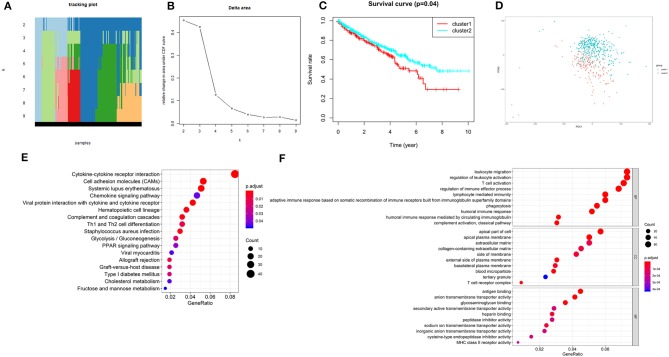
Overall survival of ccRCC patients in cluster 1 and 2 subgroups. **(A)** The tracking plot for k = 2 to k = 9. **(B)** Relative change in area under CDF curve for k = 2–9. **(C)** Kaplan-Meier overall survival (OS) curve for ccRCC patients in cluster 1 and 2. **(D)** Principal component analysis of the total RNA expression profile. ccRCC in cluster1 are marked in red. **(E,F)** Functional annotation of the genes with different expression in the cluster 1 using KEGG pathway **(E)** and GO terms **(F)**.

According to the expression similarity of the 2 m^6^A RNA methylation regulators (METTL14 and METTL3) identified above as the powerful independent prognostic factors, k = 4 could be the optimal choice with clustering increasing from k = 2–9 (Figures 6A,B). However, we observed that only when k = 2, the interference between subgroups was minimal (Supplementary Image 1). Hence, k = 2 was used for consensus clustering analysis and two subgroups named cluster1 and cluster2 were identified. We found that patients in cluster1 had a significantly shorter overall survival (OS) than those in cluster2 (Figure 6C).

**Figure 7:**
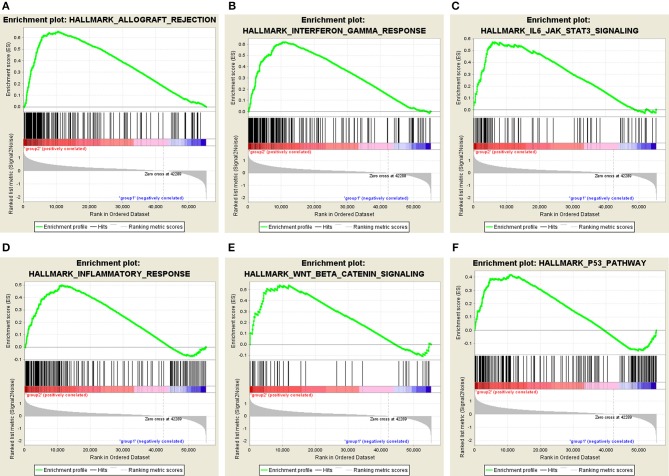
GSEA showed that genes with higher expression in cluster1 were enriched for hallmarks of malignant tumors: **(A)** allograft rejection, **(B)** interferon gamma response, **(C)** IL6/STAT3 signaling, **(D)** inflammatory response, **(E)** Wnt/β-catenin signaling, and **(F)** P53 pathway.

Moreover, we performed principal component analysis (PCA) for comparison of the transcriptional profile between cluster1 and cluster2. The result suggested that there was a significant distinction between the two subgroups (Figure 6D). Then, the genes significantly upregulated (fold change> 2 and *p* < 0.05) or downregulated (fold change < −2 and *p* < 0.05) were selected for gene ontology (GO) and Kyoto Encyclopedia of Genes and Genomes (KEEG) pathway analysis. The results of KEEG analysis showed that differentially expressed genes were mainly enriched in malignancy-associated pathways, including “cytokine-cytokine receptor interaction,” “cell adhesion molecules (CAMs),” “systemic adhesion lupus erythematosus,” “chemokine signaling pathway,” “viral protein interaction with cytokine and cytokine receptor,” “complement and coagulation cascades,” “th1 and th2 cell differentiation,” and so on (Figure 6E). GO analysis results were associated with cancer-related biological processes, including “leukocyte migration,” “regulation of leukocyte activation,” “regulation of immune effector process,” and so on (Figure 6F). Next, the gene set enrichment analysis (GSEA) was also performed and the result suggested that the malignant hallmarks of cancer, containing allograft rejection (NES = 1.94, normalized *p* = 0.008, FDR *q* = 0.070), interferon gamma response (NES = 1.81, normalized *p* = 0.038, FDR *q* = 0.134), IL6/STAT3 signaling (NES = 1.77, normalized *p* = 0.024, FDR *q* = 0.125), inflammatory response (NES = 1.74, normalized *p* = 0.032, FDR *q* = 0.117), Wnt/β-catenin signaling (NES = 1.68, normalized *p* = 0.038, FDR *q* = 0.113), and P53 pathway (NES = 1.57, normalized *p* = 0.041, FDR *q* = 0.177) had significant correlations with the cluster1 (Figure 7). These results above showed the two clusters identified based on the two powerful independent prognostic factors were closely associated with the malignancy of ccRCC.

## Discussion

CcRCC is one of the most popular common form of renal cancer worldwide. There are no effective therapeutic strategies for ccRCC patients with advanced stage or metastasis and the rate of 5-years disease-free survival in patients with metastasis is only 12%. M^6^A RNA methylation was demonstrated to be closely associated with tumorigenesis and overall survival (OS) in patients with renal cell carcinoma (37). In the present study, the expressions of m^6^A RNA methylation regulators are also highly correlated to the tumor progression and prognosis in ccRCC. Firstly, 10 of 13 m^6^A RNA methylation regulators were identified as playing important roles in the development of ccRCC, including ZC3H13, METTL14 and YTHDF2, FTO, ALKBH5, WTAP, METTL3, YTHDC2, KIAA1429, and RBM15. We also found that the expression of FTO, METTLE14, YTHDC1, ZC3H13, and KIAA1429 was negatively related to grade and stage classification. Next, a prognostic risk signature of two identified m^6^A RNA methylation regulators was retrieved based on the data from TCGA. Then, according to the prognostic risk signature, patients with ccRCC were assigned into high-risk and low-risk groups. Then, two ccRCC subgroups (cluster1 and cluster1) were also identified by consensus clustering according to the expressions of the 2 m^6^A RNA methylation regulators (METTL14 and METTL3), which were selected for construction of the risk signature. We found that the cluster1 and cluster2 not only affected the prognosis of ccRCC patients and clinicopathological characteristics but were also associated with the cancer-related pathways, pivotal biological processes and hallmarks in ccRCC.

Recently, more and more studies have suggested that the abnormal expression of the m^6^A RNA methylation regulators is involved in human cancer. The writer WTAP was reported to be a oncogene in different cancers, such as AML (38), glioblastoma (39), and pancreatic ductal adenocarcinoma (40). The eraser ALKBH5 was observed to be up regulated in glioblastoma stem-like cell (GSCs), leading to the initiation and development of glioblastoma (41). Interestingly, the reader YTHDF2 was suggested to facilitate the migration of prostate cancer *in vitro* (42) but inhibit invasion and migration in pancreatic cancer (43). These researches showed that alteration of the m^6^A RNA methylation regulators was observed in cancers and the same regulator could function as diverse roles in the initiation and progression of various cancers.

As shown in the section of results, the expression of the 13 m^6^A RNA methylation regulators in ccRCC was observed to associated with different clinicopathological characteristics. In term of m^6^A RNA methylation writers, the expression of KIAA1429, ZC3H13, and METTL14 were significantly decreased in ccRCC patients with high grade and later stage. Surprisingly, KIAA1429 was observed to be up regulated in ccRCC tissue samples compared with normal tissue samples. As an m^6^A RNA methylation eraser, the expression of FTO was found to be similar to that of KIAA1429, up regulated in ccRCC patients while down regulating in ccRCC patients with high grade and later stage. This may be because KIAA1429 and FTO could exert various functions at different stage of ccRCC tumorigenesis and development. KIAA1429 is also named as vir-like m^6^A methyltransferase associated or VIRMA and was reported to promote the proliferation, migration and invasion of HCC by regulating mRNA methylation levels of ID2 in cell lines (44). Another research also showed that KIAA1429 was significantly up regulated in seminomas, while down regulated in non-neminomatous tumors (45). FTO was reported to be significantly associated with bad prognosis in ccRCC patients and its prognosis value increased as the increase of stages (46). Low expression of ZC3H13 was associated with the progression of colorectal cancer by inactivating Ras-ERK signaling pathway (47). A recent study reported that METTL14 could mediate P2RX6 mRNA and protein level, promoting renal cancer cells migration and invasion via ATP-induced Ca2^+^ influx modulating ERK1/2 phosphorylation and MMP9 signal pathway *in vitro* and *in vivo* assays (48). METTL14 was also suggested to be an inhibitor of tumor metastasis and considered as a favorable factor in HCC via mediating m^6^A-dependent miRNA processing (49). However, another research demonstrated that METTL14 functioned as an oncogene to promote tumorigenesis by mRNA m^6^A modification in leukemia (50). FTO was demonstrated to promote the proliferation, transformation and survival of acute myeloid leukemia cells *in vivo* and *in vitro* (51). As one of the two powerful prognostic m^6^A RNA methylation regulators, METTL3 mRNA and protein was reported to be downregulated in RCC tissues by quantitative real-time PCR (qRT-PCR) and western blot, which was in accordance with our results. Then, the researchers also found that low level of METTL3 was associated with larger tumor size and higher histological grade *in vivo* study, and could promote RCC cell proliferation, migration and invasion function and induced G0/G1 arrest *in vitro* study. In addition, significant changes in epithelial-to-mesenchymal transition (EMT) and PI3K-Akt-mTOR pathways were noted in their research. This indicated that METTL3 could exert its function via EMT and PI3K-Akt-mTOR pathways, which is worth further investigating. Moreover, the researchers found that upregulated expression of METTL3 always associated with favorable prognosis in ccRCC patients (37). Zhou et al. (34) also suggested that low level of METTL3 was correlated with some cancer-related biological processes, including adipogenesis, mTOR pathway and reactive oxygen species. In summary, dysregulation of m^6^A RNA methylation regulators were highly correlated with tumor progression and prognosis in ccRCC. These findings could also provide novel methods for the treatment and prevention of ccRCC.

Next, GO and KEEG analysis associated with m^6^A RNA methylation regulators were also conducted in this study. Several biological processes and pathways correlated with the tumorigenesis and progression of ccRCC were identified including “cytokine-cytokine receptor interaction,” “cell adhesion molecules (CAMs),” “complement and coagulation cascades,” “leukocyte migration,” “regulation of leukocyte activation,” “regulation of immune effector process,” and so on. It was known that the expression or function of cytokines, including cellular self-renewal, senescence, migration, and apoptosis was often altered in tumor tissues compared with healthy tissues (52–54). CAMs was found to promote the metastasis of head and neck squamous cell carcinoma via detaching the tumor cells from primary tumor and decreasing the intercellular adhesion (55). More and more evidences suggested that complement component and complement activated product could facilitate the growth and angiogenesis of tumor, as well as immunosuppression (56–58). A recent research (59) reported that the abnormal downregulated genes were possibly associated with the gallbladder cancer progression through the complement and coagulation cascades. Leukocyte migration was suggested to be activated in the progression of cancer (60). A study was showed that immune system was closely associated with the progression of hepatocellular carcinoma (61). Here, we revealed that the m^6^A RNA methylation regulators were involved in many biological processes and signaling pathways, suggesting their important roles in initiation and development of ccRCC.

In this study, we found that the prognostic signature obtained using 2 m^6^A RNA methylation regulators (METTL14 and METTL3) had significant value in ccRCC. The ROC for risk signature identified above exhibited a satisfied prediction performance in ccRCC. Additionally, the prognostic value of signature for low grade and high grade, early stage and later stage ccRCC was also noted. High-risk Patients even with the same high grade or later stage had a significantly shorter survival time than low-risk patients. Risk signature in the present study may be benefit for physicians to more precisely estimated individualized survival prediction.

However, several limitations in this study should be acknowledged. First, the number of normal samples (72) is much less than that of tumor samples (539). It may affect the reliability of our results. Second, the present study is purely computational, future experimental and clinical data are need to validate our results. Third, some important clinical parameters of ccRCC patients, such as treatment strategy, vascular invasion and surgical margin, are not available from TCGA. Finally, patients in our study are mainly Americans and this may lead to the risk of potential selection bias.

In conclusion, our study revealed that the expression of the 13 m^6^A RNA methylation regulators are closely correlated with the malignant clinicopathological characteristics of ccRCC and are also highly related to the upregulated expression of genes enriched in the biological processes and pathways that facilitate the malignant development of ccRCC. Our findings may be regarded as important evidence for the role of m^6^A RNA methylation in ccRCC. Future experimental and clinical studies are necessary to produce a solid confirmation of our results.

## Data Availability Statement

Publicly available datasets were analyzed in this study. This data can be found here: The Cancer Genome Atlas (TCGA) database.

## Ethics Statement

This study met the publication guidelines stated by TCGA (https://cancergenome.nih.Gov/publications/publicationguidelines). All data used in the study was obtained from TCGA, and hence ethics approval and informed consent were not required.

## Informed Consent

Informed consent was obtained from all individual participants included in the study. And the present research meets the publication guidelines provided by TCGA (https://cancergenome.nih.Gov/publications/publicationguidelines).

## Author Contributions

JW, WH, and XG contributed conception and design of the study. JW and CZ organized the database. JW and CZ performed the statistical analysis. JW wrote the first draft of the manuscript. CZ wrote sections of the manuscript, contributed to manuscript revision, and proofreading. All authors contributed to manuscript revision, read, and approved the submitted version.

### Conflict of Interest

The authors declare that the research was conducted in the absence of any commercial or financial relationships that could be construed as a potential conflict of interest.

## Supplementary Material

Supplementary Image 1 Consensus clustering matrix for k = 2 **(A)**, k = 3 **(B)**, and k = 4 **(C)**.

Supplementary Table 1 TCGA sample IDs and RNA-Seq information.
