# Supplementary figures and images for: Effect of m6A RNA Methylation Regulators on Malignant Progression and Prognosis in Renal Clear Cell Carcinoma
Source: Front Oncol. 2020 Jan 24;10:3. doi: 10.3389/fonc.2020.00003 (PMC6992564; doi:10.3389/fonc.2020.00003)

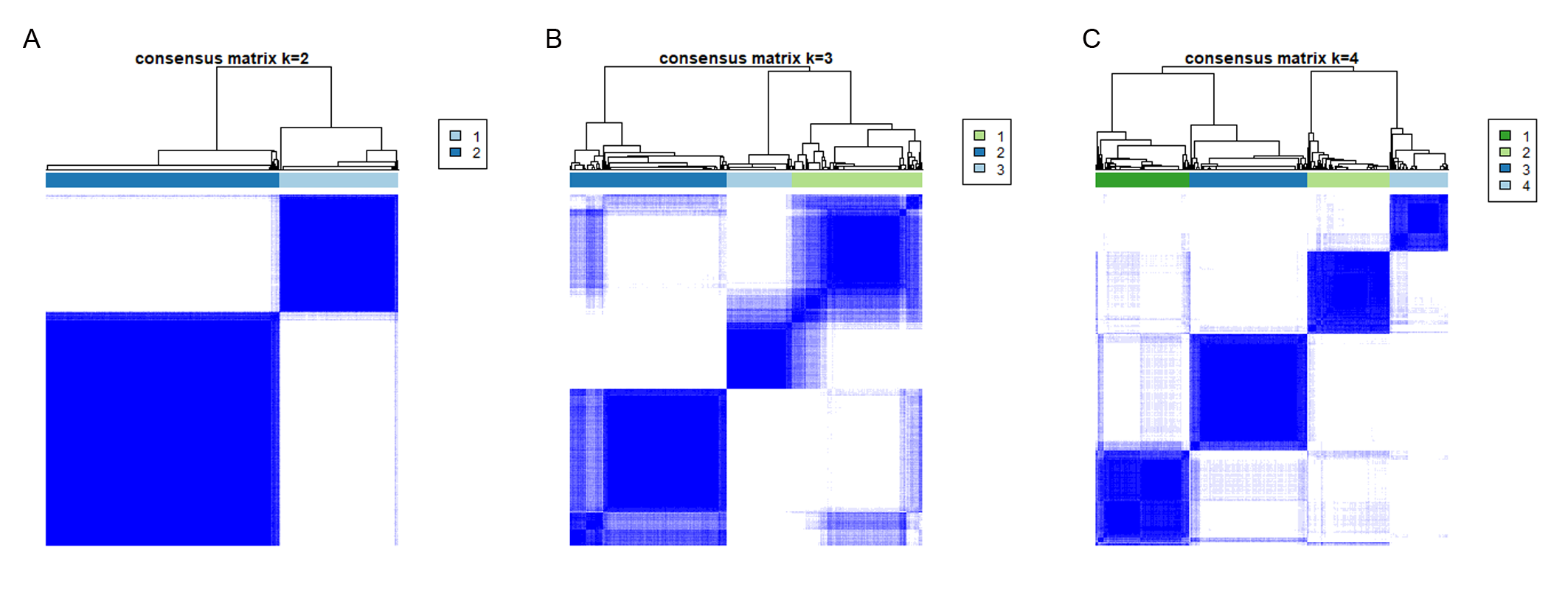

Supplement: Supplementary Image 1 — Consensus clustering matrix for k = 2 (A), k = 3 (B), and k = 4 (C). [file Image_1.TIF]
